# Informing a culturally appropriate approach to oral health and dental care for pre-school refugee children: a community participatory study

**DOI:** 10.1186/1472-6831-14-69

**Published:** 2014-06-13

**Authors:** Pam Nicol, Arwa Al-Hanbali, Nigel King, Linda Slack-Smith, Sarah Cherian

**Affiliations:** 1 School of Paediatrics and Child Health, Faculty of Medicine, Dentistry and Health Science, M561 University of Western Australia, Perth 6009, Western Australia; 2 School of Dentistry, M512, University of Western Australia, Perth 6009, Western Australia; 3 Department of Paediatric & Adolescent Medicine, Princess Margaret Hospital, GPO Box D184, Perth 6840, Western Australia

**Keywords:** Early childhood caries, Refugee experience, Cultural

## Abstract

**Background:**

Pre-school children in families of recently settled refugees often have very high rates of early childhood caries (ECC). ECC is associated with a high level of morbidity and is largely preventable, however effective culturally appropriate models of care are lacking. This study aimed to provide a deeper understanding of the refugee experience related to early oral health by exploring pre-school refugee families (i) understanding of ECC and child oral health, (ii) experiences of accessing dental services and (iii) barriers and enablers for achieving improved oral health. The knowledge gained will be critical to the development of effective early oral health programs in refugee children.

**Methods:**

Community based participatory qualitative methodology using focus groups of resettled refugee families and community refugee nurse interviews. A community reference group was established and a bi-lingual community research associate was employed. Transcripts were analysed for thematic content using NVivo software.

**Results:**

There were 44 participants: eight focus groups (nine countries of origin) and five interviews. Emergent themes were (i) the major influence of parents’ previous experience, including their beliefs about deciduous (baby) teeth, traditional feeding practices and poverty; and a consequent lack of understanding of the importance of early oral health and early dental caries, (ii) the burden of resettlement including prioritising, parenting, learning about new foods and how to assimilate into the community, and (iii) refugees’ difficulties in accessing both information and dental services, and the role of schools in addressing these issues. An Opportunities for Change Model was proposed.

**Conclusions:**

The main implication of the study is the demonstration of how enhanced understanding of the refugee experience can inform improvement in early oral prevention and treatment. The community participatory methodology of the study provided a basis for cross-cultural understanding and has already assisted in translating the findings and raising awareness in the provision of targeted refugee oral health services.

## Background

Children in the lowest socioeconomic groups are known to have worse oral health than those in the highest stratum [1]. In the Australian context of this research, data show the rate of mean number of decayed, missing and filled deciduous teeth (dmft) of children from the lowest socioeconomic status areas are about 70% higher than for those from the highest socioeconomic status areas [2]. In a recent prospective Western Australian (WA) study of 105 refugee pre-school children following resettlement in Australia, 77% of the families lived in a suburb in the lowest two socioeconomic quartiles (Socio-Economic Indexes for Areas (SEIFA)) [3]. Of these refugee children, (mean age three years), 62% had at least one tooth with untreated dental caries (decay) and they had a mean dmft of 5.2 (SD 4.1), compared to overall Australian children aged five to six years who had 41.3% with untreated dental caries and a mean dmft of 2.0 [4].

However, socioeconomic disadvantage is just one of the complex factors that interact to contribute to poor oral health [1]. Many refugee children already have severe dental disease when they enter Australia, often progressively worsening after resettlement [5]. Refugee families face many barriers in accessing appropriate health care post-resettlement [6] and are less likely than non-refugee children to access dental health services [5]. In the WA study referred to previously, in a 12 month period less than half of the pre-school refugee children with untreated decay saw a dentist, and, compounding the difficulties they faced, 45% had severe disease that required costly specialist dental management [4].

However, despite this, many health professionals lack a clear understanding of these barriers and of refugee families’ perceptions of oral health in their children. The fundamental premise of this research was that in order to improve the dental health care of the children we first need to understand refugees’ perceptions of oral health and explore their experiences of dental health services. In this way, the barriers and enablers with regard to their utilisation of services in Australia could be identified. The study focused on refugees in new and emerging communities. These are people who are sometimes identified as “high need clients”; due to the length of time spent in refugee camps, their lack of personal support networks in Australia and the additional assistance and resources needed to address the settlement challenges they face. Some communities may share these characteristics for up to ten years [7]. Australia uses the United Nations High Commission for Refugee (UNHCR) definition for a refugee as someone who “owing to a well-founded fear of being persecuted for reasons of race, religion, nationality, membership of a particular social group or political opinion, is outside the country of his nationality, and is unable to, or owing to such fear, is unwilling to avail himself of the protection of that country [7]”.

Refugees from new and emerging communities are not homogenous. There are some comparative studies on the effect of different cultures on oral health care for young children which may help identify why the refugee families are not accessing the services that are available [8]. It has been suggested that improvements in health outcomes within a multicultural population may be attained by identifying the knowledge and behaviours that offer most opportunity for improvement in clinical outcomes [9-11]. For example, it has been shown that the level of behavioural and psychological acculturation within the Vietnamese population living in Australia was an important intervening variable in three outcome measures of oral health. The middle level of acculturation had the worst outcomes. The authors suggested that a reason for this was that cultural belonging was a protective factor and the middle level group may not fit into either culture [9]. Other studies have demonstrated that cultural or parental perspectives affect oral health in refugee populations [12-18]. Nevertheless, there is still minimal qualitative data on the effect of traditional practices and of transit and resettlement experience on the behavioural and psychological adaptation required for refugees to value and adequately manage early childhood oral health.

Demonstrations of “the capacity to manage (their own) health and wellbeing have become central components of citizenship in post-industrial societies [19]”. Measuring this capacity has led the concept of health literacy. “Health literacy” refers to accessing, understanding and using information to make health decisions [20,21]. For example, in a Canadian study of immigrant women participants said to have high health literacy asked more specific questions about diet-related cancer prevention than women with low health literacy. The authors concluded that, to optimise their understanding, both groups needed specific, culturally sensitive information at the right level for their current level of literacy [22]. Similarly, a study of Australian migrant women concluded that new immigrants require a staged introduction to new food specific information [23]. However, despite the importance of health literacy levels, strategies for assessing these by dental teams remain largely unexplored [24].

Others have suggested that more understanding of the construct of health care empowerment, i.e. an increasing involvement of patients and clients in their own health care, is needed. Assuming people have a desire for choice and control over their own health, they can become engaged, informed, collaborative, committed and tolerant of uncertainty [25,26]. The construct is influenced by the interplay of cultural, social, and environmental factors, personal resources and intrapersonal factors. Critically, the refugee population cannot be at this level of empowerment whilst experiencing the stress of resettlement. Therefore, service providers need to consider the refugee experience and its impact on health behaviours of this population [8,27].

The present study explores the perceptions of new and emerging refugee communities in Western Australia with regard to their experiences of dental health services, in order to increase understanding of and improve oral health literacy within these communities. It is intended to provide valuable information for the planning and delivery of culturally responsive pre-school oral health and oral health promotion strategies.

### Aims

The purpose of this study was to explore how humanitarian entrant refugees understand and make sense of good oral health in pre-school aged children. The three research questions that guided the study were:

a) What are refugee carers’ knowledge of and understanding of oral health in children, with specific reference to the causes, impact and prevention of early childhood caries (ECC)?

b) Are there any issues with current access to services for the treatment of ECC?

c) What are the main barriers and enablers for these refugee families to achieving adequate oral health in early childhood?

## Methods

### Study design and methodology

The methodological approach used for this research study was a community-based participatory qualitative study, using focus groups supplemented by individual interviews. This approach was chosen to promote engagement and capacity building strategies in the community through participants’ sharing of experience and expertise, thereby cultivating community ownership of the research outcomes [28-30].

Community involvement was fostered, firstly, by the employment of a research assistant from the community, who was trained in basic qualitative research techniques by an experienced research team facilitator, and, secondly, by the establishment of a community reference group (CRG).

The sixteen CRG members included bilingual representatives from four new and emerging communities, refugee service agencies, health promotion and community refugee health professionals. The CRG worked with research team members to develop the terms of reference for the study, named the study “Beginning with Healthy Teeth” (BHT) and advised on questions, recruitment, methodology and translation of findings in a culturally appropriate way [31].

### Data collection

The sampling was purposive. The inclusion criteria were parents, grandparents, or guardians of humanitarian entrant or asylum seeker children aged less than five years from new and emerging refugee communities. Recruitment of focus group members was through invitation by CRG bicultural workers and community representatives. An invited sample of community refugee child health nurses was interviewed to enhance the understanding of refugee oral health issues.

Focus group interviewing was used because this has been shown to facilitate gathering of richer data, and to be culturally safer, than individual interviews in migrant and refugee women [31,32]. Focus groups also encourage storytelling, which is central to participatory action research [29]. At the beginning of the focus group, participants were asked to respect one another’s confidentiality and opinions.

For the focus groups a semi-structured technique was used. The questions were based on participants’ ideas and experiences related to pre-school oral health beliefs, utilisation of dental services, and early feeding and family nutrition practices pre- and post-settlement. Focus groups were conducted in the participants’ own languages and translated into English by interpreters during the focus group.

The audio recordings were transcribed verbatim into English and all identifying data were removed at this point. Data collection continued until saturation, which “occurs when researchers sense they have seen or heard something so repeatedly that they can anticipate it”, was reached [33]. A research diary was kept by the researchers conducting the focus groups to provide evidence of the research process and enable reflection on personal positions and biases that could influence the analysis [34].

The demographic data collected from the interview groups included education level, occupational status, ethnicity, transit country, years since arrival in Australia, language(s) spoken and age group.

### Data analyses

Demographic data describing the participants were analysed using SPSS Statistical Software Version 19. Iterative inductive thematic analysis was used to code, sieve, group and interpret the data and elucidate themes [34] utilising NVivo (Version 9) computer assisted qualitative data analysis software of the combined qualitative data transcripts and written notes. Two of the researchers analysed data independently at each stage of the process, and discussed similarities and differences before the next iteration. Data were tagged by ethnicity, and for common themes and differences and questions for further analysis. The interpretation of the data was then reviewed by the researchers for clusters by ethnicity and for cultural soundness by community representatives of each ethnic group. Finally the transcripts and analysis were reviewed, common themes and differences integrated, and findings compiled. The draft report was presented back to the CRG by the researchers at the completion of the study for their recommendations.

Approval was obtained from the Child and Adolescent Health Service Human Ethics Committee (Princess Margaret Hospital for Children #2010EP) and the University of Western Australian Human Ethics Research Committee (RA/4/1/5640). Permission was obtained from each participant for audio recording of the focus group or interview. Interpreters were used for all focus groups and for ensuring the signed consent was understood.

## Results

### Focus groups and interviews

Data were collected from December 2012 to February 2013. Eight focus groups and five community health nurse interviews were completed. The total number of participants was 44, with focus groups numbers ranging from four to seven.

### Demographic characteristics

Refugee participants (n = 39) were from nine different countries of origin (Table 1), and were mostly mothers (95%) between 30-39 years of age (54%) who had been in Australia for a median time of four years. The main transit country was Thailand (33%). The most common languages were Karen (36%) and Arabic (31%). Twenty percent had professional qualifications but none worked in a professional capacity in Australia.

**Table 1:** Countries of origin of focus group participants

Country of origin	Frequency (percent)
Burma	16 (41)
Iraq, Kuwait (Middle Eastern)	9 (23)
Sudan	5 (13)
Afghanistan	3 (8)
Burundi	2 (5)
Democratic Republic of Congo	2 (5)
Rwanda**,** Nigeria*	1 each (5)

*Not classified as a refugee country.

NB Because there are so few participants from the African countries apart from Sudan, they are referred to in the text by continent rather than nationality to preserve their anonymity.

### Themes

Three main themes emerged:

o Parents’ past experience

o Resettlement issues

o Enablers and barriers to accessing dental services

### Parents’ past experiences

#### Parents’ previous experience with early dental services

The principal factor affecting the refugees’ attitudes to their children’s oral health care was the context of where they had lived, i.e. rural villages, towns or refugee camps.

In rural areas of all countries of origin, it was common not to have a dental clinic. For example, one mother said:

Well in (rural) Sudan we don’t go anywhere; even the midwife comes to our home to deliver the baby.

Where available, clinics were staffed by “technicians” not dentists and were focussed on removing carious deciduous teeth rather than providing preventative services.

Participants reported that some other countries, such as Nigeria, did have good preventative services. When available these services were expensive and only accessible to people who lived in cities and could afford them. Iran and Iraq were identified as having very good and affordable oral health services, with children’s dental health promotion messages on television, although the services in Iraq had been disrupted by war. Access could also be restricted for other reasons. For example, in Kuwait, Bedouins could not access any government services as they were not recognised as citizens by the State.

#### 2. Beliefs about deciduous (first) teeth

Most participants were not concerned about their children’s deciduous teeth, which they reported as *going to fall out anyway.* Dealing with early dental caries was commonly done without a dental clinic visit, e.g. a Sudanese mother said:

if the tooth is not good or loose we just pulled out using a thread, we never took our babies to dentist.

A Burmese mother explained there was a practice when the deciduous teeth were coming through and it was “a bit sore”, they dug the tooth out.

Overall, participants noted few impacts of early caries. Those that were mentioned were *bad smell, makes them bad tempered, not happy, don’t eat well*.

#### 3. Poverty in transit camps

Several Burmese people from refugee camps said there *are some toothpaste and brushes in camp shop but very expensive and we do not have the money, so we use salt*.

Refugees who had lived in transit camps for extended periods of time also had a very different experience of food security. A Sudanese mother explained:

Yes I used to live in the camp for 11 years the situation was very hard, the food was very little, and we only eat lentil and we have only one cup of oil per family each month and a bit of flour.

When food was available refugee families had often lacked the money to provide more than a minimal diet for their children, e.g., in Thai border camps infant formula was replaced by condensed milk because the formula was so expensive. Where even this was too costly, mothers used honey and water mixtures.

#### 4. Traditional early feeding practices

Prolonged breastfeeding (greater than 12 months) on demand and co-sleeping were common across all the cultural groups. However, exclusive breastfeeding was not always practiced. Some participants had used both the bottle and the breast to feed, e.g. an Afghani mother said: *We breastfeed up to two years, and yes it is common in Afghanistan to mix the breast milk and formula, child sleep with bottle.* In other cultures, use of the bottle was due to the need to work, although for middle and upper classes participants deemed a bottle feeding trend was a response to advertising. African mothers tended to have used more exclusive breastfed than others unless they were working: For example in the Sudan:

We breastfeed girls up to 18 month and boys up to two years, because the girls is more bigger than the boys, other believe that if you breastfeed the boy two years he will be less smart so we only feed him up to 18 month

The introduction of solid foods into an infant’s diet was generally according to the World Health Organization guidelines to start at six months, although participants identified considerable disparity in the nature of the food. For example, the Burmese traditional food for weaning was soft boiled egg, banana, kongi (rice), fine minced meat, potatoes and carrots. Karen mothers first gave rice, very soft cooked, and squeezed through cloth so it became starch, followed by banana and papaya. A community health nurse spoke about a pre-mastication practice utilised by Karen mothers from about six months of age. Additionally, the excess of juice and/or sweet bubble tea ingestion was noted in Karen communities by a community nurse.

African first foods were less carbohydrate rich. For example, an African mother explained:

I used to mix the breast milk and formula, but at six months, start pureed fruits. Our porridge is a mix of sorghum, maize, wheat, soya and milk. No, don’t usually sweeten. May start having tea after one year, sometimes I use honey, just a little bit of sugar, half a small spoon to make it flavour.

Traditional ways of caring for teeth included rubbing with charcoal (Burma), chewing betel nut and rocks (Ethiopia), miswak (sticks) (Afghani), salt and green skin from walnut fruit or nuts (Afghani). Traditional pain relief methods for toothache included rubbing the gums with clove oil (Burma) or hot date pulp (Sudan). These methods were passed on through grandparents. The loss of grandparenting due to family separation was also lamented by some participants, because grandparents were an important part of learning and sharing of parenting.

### Resettlement issues

#### 1. Parenting in a new culture and learning about new foods and water

Participants reported that where they had experienced problems in providing for their children during transit, they then had difficulties on resettlement where food appeared plentiful, e.g. an African mother said:

When we first arrive, we do not know, colour and everything very tempting, so we give to children, children take to school, but teachers tell us it is not good for children, so now we know it is not good.

The parents’ stories had a common theme that the parents wanted to compensate for their children’s difficult start in life by giving them what they perceived as good things in Australia. Several participants said that if their children asked for anything, they found it difficult not to buy it for them. However as their understanding of foods in Australia developed, their emotional need to do the best for their children often led to conflict as children’s demands became influenced by advertising, television (TV) programs, supermarket displays and peer group pressure. Most participants said they were aware of the effect of TV commercials on children’s choices but that their children listened to them less as they got older and watched more TV. Reinforcing the effect of TV, the ready availability and display of foods in supermarkets also led to conflict as parents tried to make healthy purchases. An African father explained:

When they are growing up in camp, if we said we don’t have money, they would understand, here, they know you have the money. They see everything; they know we can’t discipline them.

Most of the parents said they were struggling with parenting and particularly with disciplining their children during resettlement. For example, a Karen mother said:

Sometimes my daughter likes to drink soft drink, she likes so much, and we had to hide it. Now she only drinks milk energy drink [name removed], I don’t know how to stop it, and she drinks 4-5 packets a day. She doesn’t like anything else after stop breastfeeding at one and a half years.

Exposure to soft and sweet drinks before arrival in Australia varied for different nationalities. Soft drink was familiar to Middle Eastern and Afghani refugees but for some African participants it was foreign prior to their arrival in Australia. The Burmese had other traditional sweet drinks: a Burmese mother who had lived in a city described the tea shops that sold sweet drinks such as tea sweetened with condensed milk which the children loved, and sugar cane juice. However, participant from a Karen rural area explained these were unavailable in their villages. Regardless of their previous exposure, most participants were aware that soft drinks can cause caries, but their consumption was nevertheless common in their homes.

Soft drink was sometimes used as the drink of choice because of a dislike of the tap water in Australia, with the result that children on resettlement were not consistently accessing fluoridated water. For other participants this was a consequence of the experience of “bad” water in refugee camps and/or their country of origin. This fear of drinking tap water persisted despite local education regarding drinking water safety. Others disliked the smell and taste of the tap water, and many filtered their water because, as a Karen mother said:

at home, we were used to clean cool water from a ground spring.

Participants were mostly unaware of fluoride and its role in promoting healthy teeth, and a few expressed a fear that it was carcinogenic. Many agreed that if they could afford it, they would buy bottled water, which some saw as a sign of wealth.

#### 2. Fitting in and appearances (acculturation)

A desire to look good in order to fit into Australian society influenced ideas about personal appearance. For all participants, healthy teeth were part of this. In some cases this reinforced previously held attitudes. For example, a Sudanese participant reported that:

*Our practice in our country if you take out your teeth, if you eat something you feel ashamed, you can’t open your mouth. Like to have good teeth, it is important to us*. Africans were generally proud of their strong teeth. Commented one man: *When we were in the playground, around 17 years old, we used to say among the young men, to get a nice lady for wife, we have to check her teeth first [laughing].*

Parents reported that Australian dental services had often encouraged them to look after their children’s teeth. For example, a Burmese mother was pleased because:

I took my child to the dentist … he saw pictures of rotten teeth on the wall, so he said he didn’t want that so he now brushes regularly.

Another said:

*Only when she (my daughter) was told by the dentist that the second teeth may not form properly and that she wouldn’t be as beautiful, did I think of brushing and care of the first teeth as important* and *when the dentist explained this teeth should be kept until the adult tooth comes out, otherwise she has a very ugly teeth, we know even though my daughter won’t know but we knew, so every time I see her ugly tooth, it would be because of me*.

Dentists could also be influential in encouraging children to adopt healthy oral health care habits, as an African father explained:

So sending children to a dentist will help us parents. You can get advice, if the specialist tells the children not to do something, they will listen; they won’t listen to parents.

#### Resettlement priorities

For refugee families, the difficulties of resettlement were identified as a major barrier to understanding and use of dental services. Social issues, especially housing, food, transport and mental health tended to be the families’ main priorities. Consequently, the community refugee nurses in addressing those priorities had little time for dental and nutritional education. Additionally, participants cited the transience of the families as they sought permanent housing contributing to a lack of continuity of services.

Community nurses all commented on the poverty they saw families experiencing:

*I feel for them, life is not easy, when 80*% *of your weekly money goes on rental. How do you justify that? We understand it is better to keep them healthy, but it is a huge mind-shift for them to take your child to the dentists when there is no problem, and pay money for it [emphasis].*

Another said:

Mum said she is about to move again. Mother said she doesn’t want to pay that for a two year old, four rotten teeth in front; they are not painful but are smelly. There’s an opportunity lost. She can’t afford to have another member of family suffer.

### Health promotion issues

#### 1. Access to information

Appropriate written information for the refugees was considered to be scarce. All participants said they could not remember receiving any information about food in Australia when they first arrived but they would have liked it. One Karen mother laughed when asked if the community health nurse spoke to her about food and dental health:

Yes, but they do not talk about the teeth. They give you a whole stack of papers; I don’t know what it all is [laughing].

Although the parents wanted information to take away, it was important for them that the language was simple and basic, with short sentences that were easy to translate, limited use of medical terminology, use of pictures and inclusion of traditional foods. Several were prepared to help with translation to produce useful educational materials.

#### 2. Role of schools

A consistent emergent theme was the important role of schools in influencing children. In this context “school” referred to both pre-school and primary (elementary) school, as the experience and education of older children could affect the rest of the family. The schools’ role was not only through teachers, but also through school services, peer groups and the provision of information to parents. A community nurse explained the important role of schools in nutrition:

*Healthy eating is important in school education. I am so proud of one teacher, she inspects their lunch at pre-primary every day, and when I look, the kids have very good lunches.* However, the children *see the rest of the kids at school have boxes of juice and chips, so they think it must be better because Western kids know better.*

Additionally, traditional foods could create peer pressure difficulties for the children:

*If she took traditional food, she would be embarrassed and not going to open it,* said an African participant.

#### 3. Educational materials and training

The community nurses also commented on the lack of suitable educational materials. They provided further insight into some problems with current pamphlets. These included a focus on Western-style lunches, and lack of specific reference to different traditional foods, lack of practical advice about keeping food safe (e.g. refrigerating chicken), and insufficient use of appropriate pictures. There was also an apparent lack of understanding that some families, in addition to being unable to read English, were illiterate in their primary language and some had had little or no schooling.

The community nurses, who were aware of the daily challenges facing the families, expressed a need for preschool oral health programs to be developed to take into account the cultural background of the refugees. One commented that:

*understanding of where these people come from, their experience, and their customs, to get a little insight to relate education in a way they understand and make it human*. Another said:

*There is a lack of understanding that you have to treat some groups differently in order to treat them equally because they are disadvantaged more than the average people in Australia*.

Reinforcing this recognition of disadvantage, a refugee participant from Burma said:

Recommendation for this study would be for the government to subsidise treatment for preschool teeth.

Some of the community nurses voiced a need for themselves *to be upskilled to offer better nutrition education, particularly around maintaining traditional diets.* They were aware that translation of information into behaviour change was a complex matter and there were many reasons why it failed to happen. A community nurse provided an example of how a tradition - of offering sweet foods as hospitality to guests – could be a barrier to change:

It is interesting, when you go on a home visit, and you talk about these things (dental health) I use lots of pictures in different languages and when I have finished they offer you a can of commercial [names removed] soft drink and a bowl of lollies.

#### 4. Access to dental services

This theme of misunderstanding also emerged in discussions about enablers and barriers to accessing dental services in Australia. It applied both to the information provided to refugees and to communication between dental and health services. Despite this, there were some positive responses from parents who had experienced free or low cost services by friendly helpful staff. A Burmese participant expressed gratitude:

I had two kids when I arrived, two and a half and three years, they had really bad teeth, so I had to go to hospital, they had X-ray, and they had to take out all the teeth. And they had two crowns in, now eight and nine years, and I now take them regularly for check-up. It was a nice experience; they were kind to me.

However, more common were stories told of long wait lists and delayed treatment when a child was in severe pain and not eating or sleeping properly. This Middle Eastern mother described her experience:

…My daughter … had five or more rotten teeth… three weeks from the dental clinic visit; she became very sick with very high temperature and suffers a lot of pain. I had to take her to the emergency…they send her to do operation immediately, they pull out … total of 14 teeth, they took all the pus out and thanks God she was OK… My son had four rotten teeth, when he got the appointment after year and half, they had to pull out his teeth instead of treat them…

Participants expressed satisfaction when they experienced a friendly service including clinic reception staff, when appointments times were clear to them, and not cancelled, when they had assistance with paperwork, and an interpreter service was available. They also needed help with public transport and finding clinic locations.

The use of interpreters varied greatly among dental and health service providers and increased the information load and time required when providing information:

Send appointment letters for my son (12 years), I tick question for needing an interpreter, but I never get one (Burmese mother).

Participants reported that sometimes family members, often children, were used as interpreters.

Community refugee nurses and community child health nurses discussed their role in assisting with resettlement which may continue for up to two years. One explained that the multidisciplinary Integrated Service Centre (ISC) model helped address many of the resettlement concerns in a sustainable manner. ISC staff worked closely with the school, families and the local community. However, they had neither specific dental, nor any long term, funding. As well as scarce resources, workloads were heavy and often unmanageable, especially for such complex problems. Some nurses commented that, due to their heavy workloads, even when they could see that some information was getting lost in translation they had to ignore it and focus on the family’s immediate priorities.

The complexity of the issues the community nurses faced left some of them feeling overwhelmed and consequently feeling like they had no time to address oral health issues.

### Recommendations proposed by the community reference group

After discussion with the research team, the CRG developed the following key recommendations for refugee early childhood oral health care:

1. Develop a culturally appropriate model of care.

2. Include pre-school children in the public free school dental service.

3. Develop a WA Refugee Liaison Dental Position (RLDP).

4. The RLDP could also provide targeted community education to other refugee service agencies.

5. Community capacity building with oral health peer educators from new and emerging communities.

6. Inform medical and dental general practitioners about referral pathways and pre-school oral health needs.

7. Culturally aware health consumer representation for dental services.

8. Develop culturally appropriate educational material.

9. Promotion of the research to oral health practitioners and government funding agencies.

10. Develop national and international collaborations with a research team for further research and interventions.

### Opportunities for Change Model

**Figure 1:**
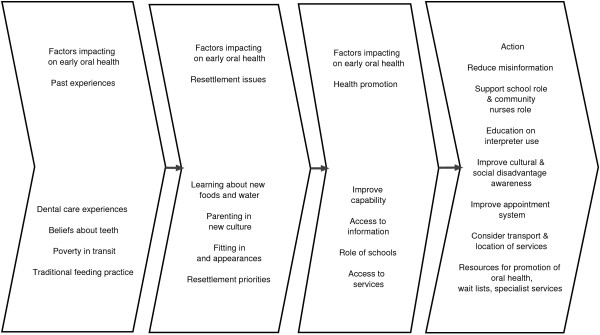
**Opportunities for Change Model.** A visual representation of the factors associated with refugee early childhood oral health.

To summarise the findings, the research team proposed an Opportunities for Change Model (Figure 1) that illustrates visually the complex interaction of factors associated with pre-school dental health in refugee children.

## Discussion

It was clear that the complex issues facing refugees resettling in Australia have led to many of them feeling overwhelmed. This is reinforced by misinformation and low health literacy in the families, leading to much misunderstanding. In addition, health professionals trying to assist the families are hampered by misunderstandings in the health system and policies and become frustrated. The recommendations developed by the CRG (see recommendations proposed by the community reference group in results section) represent opportunities to address the current situation, which can deprive refugee pre-school children of the oral health care they desperately need [6].

These outcomes of the study demonstrated that there was a general lack of understanding in oral health services of the intrinsic resettlement and logistical difficulties that refugee family’s experience. It revealed a need, when implementing an oral health initiative/model of care, for more consideration to be given to the complex interactions between the diverse past experiences of parents, a wide range of resettlement issues and conflicting priorities, and the difficulties many refugees experience when accessing Australian dental services. Our findings suggest that an understanding by providers that a more culturally sensitive approach would simplify and support access to dental services and improve treatment rates, as well as reduce the need to treat further dental caries.

This in turn requires addressing the complex interplay of causal factors that can influence oral health decisions [1]. Increasingly the importance of tailoring action to take into account the social determinates of oral health behaviour is being recognised. Our study findings support an understanding that a focus on behavioural change has not been particularly effective in improving refugee preschool oral health and that reorientation should occur to take into account common risk factors of the social determinates of health, including among others, prioritising disadvantaged groups, offering intensive and tailored support, improving accessibility and integration with other services [35-38]. Our Opportunities for Change Model, which shows the factors that influence refugee preschool health care, provides a framework for the development of an improved approach.

The development of the model was made possible by our use of a participative community methodology. From this emerged the issues that the refugee participants and health community nurses working with them perceived to be the enablers and barriers affecting the oral health care of the preschool children. In some cases, factors emerged that could be reinforced as a means of persuading parents to adopt improved oral health care strategies. For example, some African participants valued white teeth, and could easily accept a way of ensuring this. A history of breast-feeding was common, and could be further encouraged to reduce use bottle feeding and ingestion of sweet drinks. Identifying weaning foods was problematic for some Burmese mothers and help with this could reduce the consumption of sugar rich food and drinks. Other issues like fear of the (fluoridated) tap water supply could need strong evidence to overcome. Some other issues, such as the development of healthy eating habits in children with regard to sugary foods and drinks and encouragement to resist peer pressure and advertising, are a broader issue than oral health only, and may need to be addressed in a wider public health domain.

Our methodology raised community awareness of the need and benefit of early oral health. Our community reference group made ten recommendations that they identified as being likely to improve early oral health of refugees. A key to the success of these recommendations was the establishment of a refugee liaison dental position (RLDP).

The refugee liaison position would provide a link between the dental service provider, refugee community nurses and refugee families within a cultural group. The RLDP would promote links to the established network of bilingual workers within each different broad cultural group, to provide culturally specific input and access into local established social networks, such as play groups. Such a tiered approach, using established links, would reduce costs, increase capacity and provide for specific cultural needs.

The value of link workers was identified in a UK study on young black African migrants which identified that “effective health promotion communication requires more than the mechanical translation between English and the ‘foreign’ language, but must take into account client’s lived experiences, values, beliefs and cultures [39] p268”. In Australia, a review of refugee maternal child health services concluded that the “role played by bicultural workers should be recognised and utilised in a way that benefits clients and service providers” [40] (p14). In another example, the impact of primary health care delivery models for refugees in resettlement countries on access, quality and coordination found models that included bilingual staff with interpreters led to better quality of care [41]. To our knowledge, dental services have not yet widely implemented these ideas.

A further outcome of our participative community methodology was the willingness of many participants to assist with addressing the preschool oral health challenge within their community. Examples were the provision of translation services, advising on traditional foods and helping with cross-cultural understanding. Involvement at this level would be likely to further encourage local participation in preventative initiatives.

The recommendations of the CRG may be seen to fall into two broad interwoven categories: addressing lack of knowledge and understanding and addressing inadequate resources.

While the establishment of an RLDP would contribute towards the implementation of the recommendations, the dental profession can also do much to improve the service. Families in our study whose children had had a positive experience with a dental provider reported longer term improvements in family attitudes to deciduous teeth and were more likely to seek ongoing preventative care. Unfortunately, more commonly the experience was difficult, not only financially, but also because it was compounded by poor communication, transport difficulties, inconsistent use of interpreters and misunderstandings. Our study has reinforced previous research that culturally safe dental services and culturally secure dental staff, including office staff, are an important key to improving access [42,43]. The training of oral health care professionals and the appropriate accreditation protocols need to address this gap. Link workers can also help dental staff to understand resettlement difficulties and cultural understandings. More integration of mainstream refugee health services and dental services would also help, e.g., by reducing clinic attendance difficulties such as interpreter use, transport, miscommunication and multiple appointments. It may also increase the understanding that poor oral health can result in poor general health.

The need for improved education materials and communication was clearly established. This currently lacks co-ordination and would be one of the principal functions of an RLDP.

### Limitations and further research

The strong community and stakeholder engagement throughout the data collection, data analysis and recommendations phases of the project ensured the cultural appropriateness of the research as well as establishing trustworthiness of the findings [44,45].

However, a limitation was that we explored the attitudes and understanding of refugee families and of community nurses, but not of dental health services toward the needs of the families, which is a clear direction for future research. We did demonstrate that from the perspective of the refugee clients the significant difficulties these families face were often not considered in service delivery.

The study was conducted in the context of an urban area of Western Australia, with a purposive sample of volunteer participants. It is qualitative in methodology, so different perspectives may be obtained in other contexts and with other participants. Nevertheless, the outcomes may be evaluated for their transferability to other situations where the experience of the participants may be of value.

Given the multifactorial and complex interacting factors reported in this study, enhancement of oral health by improving families’ ability to manage through improved sense of control and the development of health literacy during acculturation are also worthy of further study [46,47].

## Conclusions

The participatory approach of this study has enabled a comprehensive description of the issues involved in the current failure to provide adequate dental/oral health for a cohort of preschool children that suffer high morbidity and are particularly vulnerable. The involvement of refugees themselves, as well as health care professionals provides a basis of cross-cultural understanding and hence an opportunity for all the groups to work together for the future of these vulnerable children. Action now will prevent increasing oral health problems in the future, and consequently long term saving of scarce resources will occur.

Change is already occurring with the inclusion of a dental professional in the Western Australian health care screening team for refugees. In addition, options for improved delivery of dental treatment for this group are being explored which will be inclusive of dental students; thus providing awareness of refugee issues to the next generation of dental practitioners.

Nationally, resourcing at government level and broad “higher level” issues are being addressed through recommendations to the development of the next Oral Health Plan for Australia. These issues, however, will remain challenging.

## Competing interest

The authors declare that they have no competing interests.

## Authors’ contribution

PN conceived, designed and coordinated the study, led the CRG, carried out the focus groups, performed data analysis and interpretation and drafted the manuscript. AA-H participated in the CRG, assisted with carrying out the focus groups, performed data analysis and interpretation, provided cultural interpretation and assisted with editing the manuscript. NK and LSS contributed to the design of the study, provided expert oral health advice, participated in the CRG, assisted with the development of the model and editing of the manuscript. SC conceived the study, contributed to the design, provided expert refugee health advice, assisted with the development of the model and editing the manuscript. All authors read and approved the final manuscript.

## Pre-publication history

The pre-publication history for this paper can be accessed here:

http://www.biomedcentral.com/1472-6831/14/69/prepub

## References

[B1] Fisher-Owens SA Gansky SA Platt LJ Weintraub JA Soobader M-J Bramlett MD Newacheck PW Influences on children’s oral health: a conceptual model Pediatrics 2007 120 3 e510 e520 10.1542/peds.2006-3084 17766495

[B2] Ha D Dental decay among Australian children AIHW Dental Statistics and Research Unit, Volume Cat. no. DEN 210 2011 53 Canberra: AIHW

[B3] Australian Bureau of Statistics. 2033.0.55.001 Census of population and housing: Socio-Economic Indexes for Areas (SEIFA), Australia - data only 2006 [http://www.abs.gov.au/AUSSTATS/abs@.nsf/DetailsPage/2033.0.55.0012006?OpenDocument]

[B4] Nicol P Anthonappa R King N Slack-Smith L Cirillo G Cherian S Caries burden and efficacy of a referral pathway in a cohort of pre-school refugee children Aust Dent Jin press 10.1111/adj.12269 25721281

[B5] Davidson N Skull S Calache H Murray S Chalmers J Holes a plenty: oral health status a major issue for newly arrived refugees in Australia Aust Dent J 2006 51 4 306 311 10.1111/j.1834-7819.2006.tb00448.x 17256304

[B6] Davidson N Skull S Calache H Chesters D Chalmers J Equitable access to dental care for an at-risk group: a review of services for Australian refugees Aust N Z J Public Health 2007 31 1 73 80 10.1111/j.1753-6405.2007.00014.x 17333613

[B7] Office of Multicultural Interests New and emerging communities in Western Australia Government of Western Australia 2012 [http://www.omi.wa.gov.au/omi_language.cfm]

[B8] Hilton IV Stephen S Barker JC Weintraub JA Cultural factors and children’s oral health care: a qualitative study of carers of young children Community Dent Oral Epidemiol 2007 35 6 429 438 10.1111/j.1600-0528.2006.00356.x 18039284

[B9] Mariño R Stuart GW Wright FAC Minas IH Klimidis S Acculturation and dental health among Vietnamese living in Melbourne Australia. Community Dent Oral Epidemiol 2001 29 2 107 119 10.1034/j.1600-0528.2001.290205.x 11300170

[B10] Geltman PL Adams JH Cochran J Doros G Rybin D Henshaw M Barnes LL Paasche-Orlow M The impact of functional health literacy and acculturation on the oral health status of Somali refugees living in Massachusetts Am J Public Health 2013 103 8 1516 1523 10.2105/AJPH.2012.300885 23327248 PMC3640653

[B11] Willis MS Buck JS From Sudan to Nebraska: Dinka and Nuer refugee diet dilemmas J Nutr Educ Behav 2007 39 5 273 280 10.1016/j.jneb.2006.10.005 17826347

[B12] Isong IA Luff D Perrin JM Winickoff JP Ng MW Parental perspectives of early childhood caries Clin Pediatr (Phila) 2012 51 1 77 85 10.1177/0009922811417856 21903623

[B13] Naidu R Nunn J Forde M Oral healthcare of preschool children in Trinidad: a qualitative study of parents and caregivers BMC Oral Health 2012 12 1 27 40 10.1186/1472-6831-12-27 22862892 PMC3567990

[B14] Vinay S Naveen N Naganandini N Feeding and oral hygiene habits of children attending daycare centres in Bangalore and their caretakers oral health knowledge, attitude and practices Indian J Dent Res 2011 22 4 561 566 10.4103/0970-9290.90298 22124053

[B15] Willis MS Bothun RM Oral hygiene knowledge and practice among Dinka and Nuer from Sudan to the U.S J Dent Hyg 2011 85 4 306 315 22309871

[B16] Obeng CS Culture and dental health among African immigrant school-aged children in the United States Health Education 2007 107 4 343 350 10.1108/09654280710759250

[B17] Riedy CA Weinstein P Milgrom P An ethnographic study for understanding children’s oral health in a multi-cultural community Int Dent J 2001 51 305 312 10.1002/j.1875-595X.2001.tb00843.x 11570547

[B18] Chhabra N Chhabra A Parental knowledge, attitudes and cultural beliefs regarding oral health and dental care of preschool children in an Indian population: a quantitative study Eur Arch Paediatr Dent 2012 13 2 76 82 10.1007/BF03262848 22449806

[B19] Green J Health literacy: terminology and trends in making and communicating health-related information Health Issues 2007 92 11 14

[B20] Peerson A Saunders M Health literacy revisited: what do we mean and why does it matter? Health Promot Int 2009 24 3 285 296 10.1093/heapro/dap014 19372101

[B21] Frisch AL Camerini L Diviani N Schulz PJ Defining and measuring health literacy: how can we profit from other literacy domains? Health Promot Int 2012 27 1 117 126 10.1093/heapro/dar043 21724626

[B22] Thomson MD Hoffman-Goetz L Application of the health literacy framework to diet-related cancer prevention conversations of older immigrant women to Canada Health Promot Int 2012 27 1 33 44 10.1093/heapro/dar019 21421578

[B23] Williams E Harris N Understanding the nutrition information needs of migrant communities: the needs of African and Pacific Islander communities of Logan Queensland. Public Health Nutr 2011 14 6 989 994 10.1017/S1368980010002740 21029508

[B24] Schiavo JH Oral health literacy in the dental office: the unrecognized patient risk factor J Dent Hyg 2011 85 4 248 255 22309865

[B25] de Leeuw E The political ecosystem of health literacies Health Promot Int 2012 27 1 1 4 10.1093/heapro/das001 22311156

[B26] Johnson MO The shifting landscape of health care: toward a model of health care empowerment Am J Public Health 2011 101 2 265 270 10.2105/AJPH.2009.189829 21164096 PMC3020216

[B27] Lamb CE Whelan AK Michaels C Refugees and oral health: lessons learned from stories of Hazara refugees Aust Health Rev 2009 33 4 618 627 10.1071/AH090618 20166911

[B28] Hergenrather KC Geishecker S McGuire-Kuletz M Gitlin DJ Rhodes SD An introduction to community-based participatory research Rehabil Educ 2010 24 3/4 225 238

[B29] Koch T Kralik D Participatory action research in health care 2006 Oxford, UK: Blackwell Publishing Inc

[B30] Johnson CE Ali SA Shipp MPL Building community-based participatory research partnerships with a Somali refugee community Am J Prev Med 2009 37 Suppl S230 S236 19896024 10.1016/j.amepre.2009.09.036

[B31] Merry L Clausen C Gagnon AJ Carnevale F Jeannotte J Saucier J-F Oxman-Martinez J Improving qualitative interviews with newly arrived migrant women Qual Health Res 2011 21 7 976 986 10.1177/1049732311403497 21441413

[B32] Kitzinger J Qualitative Research: introducing focus groups BMJ 1995 311 7000 299 302 10.1136/bmj.311.7000.299 7633241 PMC2550365

[B33] Sandelowski M Given LM Theoretical saturation The SAGE encyclopedia of qualitative research methods 2008 Thousand Oaks, CA: Sage Publications Ltd 876 77

[B34] Gibbs G Analyzing qualitative data 2007 Trowbridge, Wiltshire: Sage Publications Ltd.

[B35] Watt RG Sheiham A Integrating the common risk factor approach into a social determinants framework Community Dent Oral Epidemiol 2012 40 4 289 296 10.1111/j.1600-0528.2012.00680.x 22429083

[B36] Lee JY Divaris K The ethical imperative of addressing oral health disparities: a unifying framework J Dent Res 2014 93 3 224 30 10.1177/0022034513511821 24189268 PMC3929974

[B37] Çolak H Dülgergil ÇT Dalli M Hamidi MM Early childhood caries update: a review of causes, diagnoses, and treatments J Nat Sc Biol Med [serial online] 2013 4 29 38 10.4103/0976-9668.107257 PMC3633299 23633832

[B38] Peterson PE Kwan S Equity, social determinates and public health programs-the case of oral health Community Dent Oral Epidemiol 2011 39 481 487 10.1111/j.1600-0528.2011.00623.x 21623864

[B39] Ochieng BMN Black African migrants: the barriers with accessing and utilizing health promotion services in the UK Eur J Public Health 2013 23 2 265 269 10.1093/eurpub/cks063 22683768

[B40] Riggs E Davis E Gibbs L Block K Szwarc J Casey S Duell-Piening P Waters E Accessing maternal and child health services in Melbourne, Australia: reflections from refugee families and service providers BMC Health Serv Res 2012 12 1 117 132 10.1186/1472-6963-12-117 22587587 PMC3424108

[B41] Joshi C Russell G Cheng I-H Kay M Pottie K Alston M Smith M Chan B Vasi S Lo W Wahidi SS Harris MF A narrative synthesis of the impact of primary health care delivery models for refugees in resettlement countries on access, quality and coordination Int J Equity Health 2013 12 88 1 14 24199588 10.1186/1475-9276-12-88 PMC3835619

[B42] Mariño R Morgan M Hopcraft M Transcultural dental training: addressing the oral health care needs of people from culturally diverse backgrounds Community Dent Oral Epidemiol 2012 40 134 140 22998318 10.1111/j.1600-0528.2012.00733.x

[B43] Charbonneau CJ Neufeld MJ Craig BJ Donnelly LR Increasing cultural competence in the dental hygiene profession CJDH 2009 43 6 297 305

[B44] Black N Why we need qualitative research J Epidemiol Community Health 1994 48 425 426 7964349 10.1136/jech.48.5.425-a PMC1060002

[B45] Pope C Mays N Reaching the parts other methods cannot reach: an introduction to qualitative methods in health and health services research BMJ 1995 311 42 45 10.1136/bmj.311.6996.42 7613329 PMC2550091

[B46] Mittelmark MB Bull T The salutogenic model of health in health promotion research Glob Health Promot 2013 20 2 30 38 10.1177/1757975913486684 23797938

[B47] Nammontri O Robinson PG Baker SR Enhancing oral health via sense of coherence: a cluster-randomized trial J Dent Res 2013 92 1 26 31 10.1177/0022034512459757 23018820

